# The role of education in cognitive functions among middle-age and older patients with untreated obstructive sleep apnea

**DOI:** 10.5935/1984-0063.20200099

**Published:** 2021

**Authors:** Ei E Hlaing, Stephanie M Clancy Dollinger, Terry M Brown

**Affiliations:** 1 University of Lynchburg, Psychological Science - Lynchburg - VA - United States.; 2 Southern Illinois University Carbondale, Psychology - Carbondale - IL - United States.; 3 Sleep Medicine Associates, LLC, Sleep Medicine - Marion - IL - United States.

**Keywords:** Sleep Apnea, Obstructive, Cognition, Education

## Abstract

**Objective:**

The goal of the current study was to examine the interaction between educational level and obstructive sleep apnea (OSA), one of the most under diagnosed sleep disorders, on cognitive functions such as verbal fluency, psychomotor vigilance, executive functions, visuospatial ability, and attention span.

**Material and Methods:**

One hundred and nine participants (47 controls and 62 untreated OSA patients) participated in the study and completed the Wisconsin Card Sorting Test, WAIS-III digit span and block design, semantic and phonemic fluency tests, and a psychomotor vigilance task. Subjective sleep and health measures were assessed.

**Results:**

In semantic fluency and visuospatial ability tasks, patients with higher education performed better than patients with lower education and controls with lower education. This difference in moderation effects was not observed for vigilance, phonemic fluency, attention span, or executive functions although education was a significant predictor for all cognitive tasks.

**Conclusion:**

Higher education may have contributed to cognitive reserve in OSA patients (but not for controls) as reflected in better semantic fluency and visuospatial ability. This benefit of higher education contributing to larger cognitive reserve in patients with OSA helped buffer the deficits for some cognitive functions but not for others. This may indicate that this buffer is not limitless because when the cognitive demand gets higher the educational buffer no longer works.

## INTRODUCTION

Cognitive skills are necessary to maintain independent living and they become especially challenging for older adults with reported sleep problems^1^. It is reasonable to expect this trend in middle aged and older adults especially with obstructive sleep apnea (OSA). OSA is defined by experiencing at least five repetitive events of complete (apnea) or partial (hypopnea) obstructions in the upper airway per hour during sleep. Airway obstruction events contribute to falls in oxygen saturation, hypercapnia (increase in carbon dioxide), and sleep fragmentation^2^. OSA affects 9% and 24% of middle-aged women and men, respectively^3^. The decrease in cognitive functions could be related to age as well as to sleep fragmentation and OSA-related hypoxia (i.e., drop in blood oxygen level due to lack of breathing). The rate of OSA is 62% (using the cutoff of 10 or more respiratory disturbance index) in those 65 years of age and older^4^. Sleep fragmentation is purported to be associated with deficits in attentional control whereas hypoxia may be associated with executive functioning deficits^5^. It is apparent that OSA becomes more prevalent with age and may be related to deficit in cognitive performance. A variable of interest that may be associated with cognitive performance in individuals with OSA is educational attainment.

An association between a college degree and health benefits has been documented^6, 7^. A weak relation between an increase in education and improved health knowledge was observed among those who attended college, but not among those whose highest educational level attainment was high school^8^. There are two theories that may help to understand the effects of a higher education on health status. One is called resource multiplication (an aggregate of higher education is related to better health) and for this theory to hold, a college degree should not be enough to offset earlier health-related disadvantages (i.e., cognitive deficits related to OSA)^6^. In other words, patients with OSA will still perform poorly (when compared to controls, individuals with OSA-negative) even if they had at least 16 years of formal education. This theory argues that in addition to higher education, other forms of resources are needed to form an aggregate of protective factors against poor health^6^. The other theory is called resource substitution, which posits that higher education compensates for background disadvantages rather than magnifying background advantages^6^. If this theory holds true, higher education should serve as a stronger buffer for patients with OSA than controls. The focus of the current study was to test which of these two theories might be supported when examining the effects of higher education on cognitive performance specifically in a group of individuals with OSA.

When OSA patients were grouped into a high (IQ=90^th^ percentile) and normal (50=IQ<90^th^ percentile) intelligence groups, high-intelligence patients exhibited comparable attention/alertness perfor mance to the high-intelligence controls despite the OSA diagnosis^9^. Patients with normal-intelligence exhibited attention decline relative to the normal-intelligence control group. It was concluded that higher cognitive reserve (based on higher IQ) served as a protective factor for patients with OSA who did not exhibit attention deficits^9^. Cognitive reserve is the resilience to neuropathological damage such as that might be related to OSA-related cognitive impairment^10^. Factors that contribute to and are often used to assess cognitive reserve are IQ, educational level, occupation, and leisure activities that challenge the brain^11^ (e.g., learning a new language or a musical instrument). Evidence indicates that cognitive reserve may be related to cognitive functioning in clinical populations including patients with multiple sclerosis^12^, breast cancer patients undergoing chemotherapy^13^, and Alzheimer’s patients^14^. Other than Alachantis et al. (2005)^9^ study, the application of cognitive reserve theory to explain the cognitive functioning in OSA patients has not been done extensively and was the focus of the current study.

Based on Alachantis et al. (2005) study^9^, we expected to find a significant interaction between years of education (indication of cognitive reserve) and condition (OSA patients vs. controls) on six cognitive measures (i.e., executive function, verbal fluency, visuospatial ability, memory, attention span, and vigilance) even after adjusting for covariates such as mental and physical health indicators. In other words, education should moderate the relationship between OSA and cognitive functioning regardless of the covariates.

It is important to explore factors that may contribute to the maintenance of high levels of cognitive performance as it is important for successful aging. Education and OSA diagnosis factors were of particular interest in the current study because the clinical implication is that the educational level may mask OSA-related cognitive deficits. The scaffolding theory of aging and cognition-revised^15^ serves as a framework to explore the role of neural resource enrichment (e.g., higher frequency of engagement in intellectual and social activities) and life course enrichment factors (e.g., high levels of education) on the maintenance of cognitive abilities in later adulthood via compensatory processes such as neural scaffolding and cognitive reserve^15^. These factors may contribute to maintenance or high levels of performance and have been of particular interest in the area of cognitive aging research^16^. The current study was designed to specifically address the role education may have in optimizing performance in executive functions, vigilance, attention span, verbal fluency, and visuospatial ability among patients with untreated OSA as compared to a control group. To our knowledge, this has not been studied among OSA patients in depth.

## MATERIAL AND METHODS

### Cognitive measures

**Psychomotor vigilance task (PVT):** the psychomotor vigilance task measures sustained attention in a study paradigm where a response is required for infrequent targets, which pop up among frequent non-targets. This task was programmed using E-Prime version 2.0^17^. On a Spin wheel, which had six color segments (red, purple, light blue, dark blue, yellow, and green), the black three-dimensional ball rotated in each segment in a randomized order. There were four possible red slots out of the 12. The participant was instructed to press the spacebar as fast as possible every time the black ball was in the red slot and to inhibit from pressing the spacebar if the black ball was in another color. Response accuracy and reaction time were recorded. The target to non-target ratio was 20:80. The black ball was in the red slot 72 times out of 360 trials. The stimulus display was 850ms. The inter-stimulus interval was randomized between 250ms and 1000ms. Any responses faster than 200ms were considered outliers and rejected. The duration of the task, including instructions and a test trial was 11 minutes. The dependent variable was hits minus false alarms (to correct for guessing). All participants had a practice run consisting of ten trials and all questions were answered to ensure they understood instructions before continuing with the task.

**The Wisconsin card sorting test (WCST):** the WCST was developed by Grant and Berg (1948)^18^ to assess abstract reasoning and ability to shift cognitive strategies in response to environmental changes. The computerized version the Wisconsin card sorting test (Version 4) was used. Participants were presented with a number of stimulus cards on the computer screen. The items on the cards differed in color, quantity, and design (i.e., shapes). The computer was programmed to match the target card by color, design, or quantity; the participant used the computer mouse to indicate which stack (out of four) the target card belonged to. Accuracy feedback was immediately provided on the screen. During the course of the test the matching rules were changed without their awareness and the time taken for the participant to learn the new rules, and the mistakes made during this learning process were analyzed to arrive at a score (e.g., perseverating errors). The test took an average of 12 minutes to complete. The outcome score used was the number of perseverating errors (i.e., errors made from response repetition or the inability to undertake set shifting), where set shifting is the changing of goals or procedures as required^19^. Other cognitive measures include the total digit span score from the WAIS III^20^ (age-adjusted based on the norm), Block design score from the WAIS III (age-adjusted based on the norm), semantic (animals, fruits, and vegetables), and phonemic fluency (M, F, and N).

### Objective sleep measures

**Polysomnography (PSG; Sandman Sleep System, Natus Medical Inc.):** an overnight polysomnography (see table 2 for anthropometric and polysomnographic profile of the OSA patients) was used to diagnose patients with obstructive sleep apnea. Patients were referred for a sleep study prior to having a surgery or because a primary care physician had referred them if they had sleep complaints. The polysomnography equipment used at the Sleep Disorder Center included 20 channel PSG recordings. A digital in-center sleep system (Sandman SD32, software 9.3 PSG system) was used to collect information from respiratory flow, oral/nasal pressure transducer with snore detection, and oral/nasal thermistor. Thoracic and abdominal respiratory effort was measured using respiratory inductance plethysmography (z-RIP) belts. Oxygen saturation was measured by pulse oximetry. Registered polysomnography technologists manually scored all sleep stages in continuous 30 second epochs according to the American Academy of Sleep Medicine Manual (version 2.2) for the scoring of sleep and associated events^21^. It is considered an apnea if the drop in the peak signal excursion is greater than or equal to 90% according to an oronasal thermal sensor, the duration is ten seconds or longer in which the drop in signal excursion is greater than or equal to 30%, and if there is a 3% or greater oxygen desaturation or the event is associated with an arousal. It is scored as a hypopnea if the peak signal excursions drop by 30% or more, the duration of the drop higher than 31% for longer than 10 seconds, and if there is a 4% or more oxygen desaturation. All participants needed at least four hours of sleep recordings to be included in the study. In summary, a submental and mental chin electromyographic (EMG) recording, a left and a right leg EMG recording as well as respiratory signal (e.g., pulse oximetry, oral/nasal pressure, and respiratory effort) were analyzed as part of a polysomnographic diagnostic sleep study.

**Apnea Link™ (Resmed Inc.):** potential controls used the portable ApneaLink ™ at home for a night (a minimum of four hours) to screen for OSA. The Apnea Link™ was used to record respiratory nasal pressure and blood oxygen level during sleep in potential control participants. The cessation of airflow was correlated with the drop in blood oxygen level to determine if apnea or hypopnea had occurred. Oxygen saturation was measured by pulse oximetry. The scoring of apneas and hypopneas was done with the same criteria as the PSG^21^. The device is intended for screening to determine the need for further follow-up clinical diagnostic testing by a physician and evaluation by polysomnography based on the test score. A study conducted in 2009 indicated that the Apnea Link™ had acceptable reliability relative to the PSG equipment^22^. Individuals with less than five events of apnea or hypopnea in an hour were included in the study as controls.

### Mental health, mood and daytime sleepiness measures

The Beck depression inventory second edition^23^ and the Beck anxiety inventory^24^ were administered to assess depression and anxiety, respectively. The profile of mood states-short form^25^ was used to assess total mood disturbance. All participants were also asked to fill out the Epworth sleepiness scale^26^, which asked for their likelihood of falling asleep in various situations, Pittsburgh sleep quality index (PSQI)^27^, which produced a global score based on sleep duration, sleep onset latency, sleep quality, sleep medication usage, sleep disturbance, daytime dysfunction, and sleep efficiency. The higher score indicates worse sleep quality. A demographic questionnaire was administered to assess comorbidities (e.g., hypertension, diabetes, and COPD), body mass index, caffeine and alcohol consumption, and frequency and duration of regular napping (see Table 1). The morningness-eveningness questionnaire^28^ was administered to determine whether each participant was tested during his or her preferred part of day.

**Table 1:** Demographic and clinical data per group and statistical information for the comparisons between groups.

Variable	Patients (n=66)	Controls (n=46)	Chi Square test (p value)
Circadian preference (chronotype)	64.5% congruent	73.9% congruent	*p>.*05
Smoking	19.7% still smoking	10.6% still smoking	*p>.*05
Sex*	43.5% males 56.5% females	19.1 5 males 80.9% females	*X^2^* (1,108)=7.20, *p=*.007
Educational Level*	38.7%college degree or higher 61.3% high school	76.6% college degree or higher 23.4% high school	*X^2^* (1,108)=15.51, *p<*.001
Years of education*	15.52 (3.05), range 9 to 26 70.5% had caffeine	18.04 (3.40), range 13 to 28 74.5% had caffeine	*t*(107)=4.06, *p*<.001
Caffeine	within the past 3 hrs of cognitive tests	within the past 3 hrs of cognitive tests	*p>*.05
Hypertension*	50.8%	14.9%	*X^2^* (1,107)=15.02, *p<*.001
COPD	13.3%	4.3%	*p>*.05
Congestive heart failure	5%	0%	*p>*.05
Arrhythmia	10%	4.3%	*p>*.05
Diabetes*	19.7%	2.1%	*X^2^* (1,94)=7.71, *p=*.005
	M (SD)	M (SD)	*t* test (p value)
Age	54.82 (8.56), range 40-81	56.60 (12.56), range 40 to 92	*p>*.05
Nap Frequency*	2.89 (2.65)	1.34 (2.28)	*t*(74)=2.6*5, p=.*01
Nap length*	60.44 min (56.70)	18.62min (28.50)	*t*(73) = 3.86, *p<*.001
Body Mass Index*	34.83 (7.73)	24.58 (3.83)	*t*(100)=8.14, *p<*.001
Epworth Sleepiness Scale*	9.45 (4.70)	7.34 (3.72)	*t*(105)=2.52, *p=*.013
Mental status (as measured by SLUM)	26.27 (2.61)	27.55 (1.80)	*p>*.05
Anxiety*	13.42 (10.65)	6.83 (7.45)	*t*(105)=3.60, *p<*.001
Depression*	14.10 (11.04)	5.74 (5.65)	*t*(103)=4.68, *p<*.001
Mood disturbance*	23.32 (26.65)	7.63(21.84)	*t*(104)=3.46, *p=*.001

Note: The asterisk* indicates statistical significance at the .05 level. Congruent indicates the participants were tested in accordance with their circadian preference (morning vs. evening). Means and Standard deviations (M ± SD). Statistical information: Chi square test for independence: *X^2^* (degrees of freedom) and independent t tests: *t* (degrees of freedom). COPD stands for chronic obstructive pulmonary disease. SLUM: St. Louis University Mental States questionnaire was used to screen for potential dementia.

### Procedure

Institutional review boards at the hospital and university approved the study. Due to clinical symptoms, potential OSA patients sought out a diagnostic sleep study (an overnight polysomnographic recording of heart rate, breathing, brain waves, and blood oxygen level) to diagnose a sleep disorder where the cost is covered by their medical insurance. Potential controls used the ApneaLink™ portable monitor at home for a night to screen for OSA via recordings of airflow and blood oxygen desaturation during sleep. They were told to refrain from consuming alcohol in the evening of the screening, however they were not instructed to refrain from prescription drugs, which may include sedatives or stimulants. Only when they had an AHI lower than five they were included in the control group. It is important to emphasize that these controls did not have any reported sleep apnea symptoms. The entire session of administering the battery of cognitive tests took an average range between 90 to 120 minutes. This study was part of a larger study. Every patient was administered the cognitive tests before they started using their own OSA treatment (continuous or bilevel positive airway pressure machines - CPAP or BiPap, respectively).

## RESULTS

The study consisted of 109 participants (47 controls and 62 patients with untreated OSA). Patients were recruited from a Sleep Disorder Center in the Midwest part of the United States, accredited by the American Academy of Sleep Medicine, and all had received a diagnosis of OSA by a board-certified physician after an in-laboratory diagnostic overnight sleep study using the American Academy of Sleep Medicine guidelines^29^. Controls were recruited from a pool of community research participants and were classified as non-apneic controls if they had an apnea hypopnea index (AHI) of less than five, based on a portable Apnea Link™ screening device (ResMed, Inc.). There were nine males and 38 females in the control group; the patient group included 27 males and 35 females. The overall sample consisted of 86% Caucasian (n=94), 8% African American (n=9), 3.7% Asian (n=4), 0.9% Hispanic (n=1), and 0.9% American Indian (n=1). The mean age of the patient group was 54.82±8.56 (range: 40 to 81), whereas the mean age of the control group was 56.60±12.56 (range: 40 to 92).

Categorical variables (e.g., condition, sex, hypertension, and diabetes diagnoses) were analyzed using a chi-square test for independence to determine if the variables were related to condition (patients with untreated OSA vs. control group), whereas continuous variables (e.g., age, years of education) were analyzed using an independent samples t*-*test to determine whether there was a significant group difference between the controls and patients (see Table 1). Both the patients and the controls were screened for dementia using the St. Louis University Mental status exam^30^. The mean number of years of formal education for patients was 15 (high school plus three years of college); the control group’s mean was 18 (high school plus six years of further education). Based on Horne and Ostberg (1976)^28^ morningness-eveningness questionnaire^27^, 73.9% of the patients and 64.5% of the controls were tested in accordance with their circadian preference, which we denoted as congruent. An association between congruent/incongruent and condition was not observed in a chi-square test for independence analysis.

Among the OSA patients, neither AHI nor the mean blood oxygen level during sleep was related to any of the cognitive measures (see Table 2). Patients with untreated OSA had higher body mass index (*M*=34.77, *SD*=7.6) than controls (*M*=24.56, *SD*=3.87), higher anxiety (*M*=12.89, *SD*=10.67) than controls (*M*=6.98, *SD*=7.5), higher mood disturbance (*M*=23.29, *SD*=26.47) than controls (*M*=8, *SD*=21.94), longer nap duration in minutes (*M*=58.41, *SD*=56.37) than controls (*M*=19, *SD*=28.7), and higher frequency of napping per week (*M*=2.85, *SD*=2.7) than controls (*M*=1.39, *SD*=2.3).

**Table 2:** Anthropometric and Polysomnographic profile of Obstructive Sleep Apnea (OSA) patients.

	Mean	Standard Deviation
Body mass index	34.84	7.73
Total Sleep Time (min)	356.98	51.66
N1, %	5.53	3.91
N2, %	65.92	14.82
N3, %	12.77	12.67
REM sleep, %	14.91	7.14
Sleep latency, min	28.76	25.80
AHI total, events/hr	32.58	20.82
Nadir Sp O_2_, %	82.11	8.65
MeanSa O_2_, %	94.21	2.11

First, each cognitive test score was correlated with twelve variables: years of formal education, age, gender, condition (patient vs. control), PSQI, ESS, BMI, depression, anxiety, mood disturbance, diabetes, and hypertension (see Table 3). Second, the variables that were statistically significant in the correlational analysis were included in the multiple regression model, while selecting the backward elimination method (aka Model 1 in Tables 4, 5, and 6). This produces the best model (i.e., Model 2) with the highest adjusted R^2^ and reduced number of predictor variables (the lowest standardized coefficients were removed by the backward method). Subsequently, all the predictor variables from Model 2 were included in the first block of the hierarchical regression using the enter method. In the second block, the interaction term was included. In order to create an interaction term for the hierarchical regression, the variable of years of education along with all other continuous variables whose means were centered (i.e., the mean was subtracted from each raw score) and the dichotomous variable (patient with untreated OSA vs. controls) were multiplied. This allows the researcher to use the interaction (i.e., a multiplicative term) as a predictor variable in the second block to test for the moderation effect of OSA. The collinearity statistic tolerance for all regression analyses were above 0.4. It was suggested that only a tolerance below 0.4 was a cause for concern^31^. The hierarchical regression determined the statistical significance of R square change if the interaction variable was significant even after accounting for the other predictor variables.

**Table 3:** Pearson Product Moment Bivariate Correlations between Cognitive Measures and Potential Predictor Variables.

	Visuospatial	Semantic	Ln Perseverating Error	Sq rt Vigilance	Phonemic	Total Digit Span
Education	.52**	.22*	-.29**	.28**	.32**	.34**
BMI	-.23*	ns	ns	ns	ns	ns
Depression	-.32**	-.23*	ns	-.24*	-.27**	ns
Anxiety	-.31**	ns	ns	-.27**	ns	-.24*
Mood Disturbance	-.29**	ns	.23*	-.22*	-.23*	-.23*
PSQI	-.27**	ns	ns	-.26**	ns	ns
ESS	ns	ns	ns	ns	ns	ns
Age	ns	ns	ns	ns	ns	ns
Condition	.23*	-.23*	ns	.25**	.22*	ns
Gender	ns	-.32*	ns	ns	ns	ns
Hypertension	-.27**	-.22**	.32**	-.26**	-.24*	ns
Diabetes	ns	.21*	.22*	-.20*	ns	ns

*p<.05; **p<.01; *** p <.001; ns = not significant

**Table 4:** The Backward Elimination Method and Hierarchical Multiple Regression to predict Visuospatial Ability.

N= 101	Model 1 (Backward Elimination Step 1)	Model 2 (Backward Elimination Step 2 with significant predictor variables)	Model 3 (with interaction term)	Tolerance
{VIF}				
Constant	11.56 (.56)	11.32 (.25)	11.19 (.31)	
Education	.36 (.08) [.45]***			
	.36 (.08) [.45]	1.9 (.10) [.24]	.41 {2.46}	
Depression	-.05 (.05) [-.17]	-.05 (.03) [-1.8]*	-.05 (.02) [-1.8]*	.86 {1.17}
BMI	-.01 (.05) [-.02]			
Condition	-.28 (.82) [-.05]			
PSQI	-.002 (.10) [-.003]			
Mood	.000 (.02) [-.004]			
Anxiety	.004 (.04) [.01]			
ESS	.02 (.07) [.02]			
Hypertension	-.31 (.67) [-.05]			
Interaction (Education x Condition)			-.34 (.14) [-.29]*	.45 {2.22}
Adjusted R2	.22	.28	.31	

Unstandardized regression coefficients; Standard Error (in parentheses); Standardized regression coefficients [in brackets]. *p<.05; **p<.01; *** p <.001; ns = not significant

**Figure 1:**
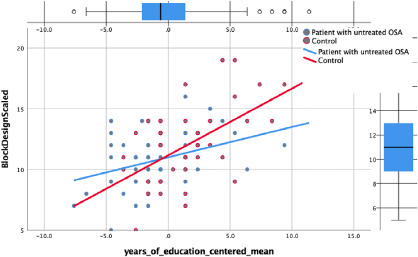
Significant interaction for visuospatial test.

**Visuospatial Ability (WAIS III Block Design):** using the backward method, the multiple regression included education, depression, ESS, BMI, anxiety, hypertension, PSQI, and mood disturbance as predictor variables in Model 1 (see Table 4). The best model (Model 2) consisted of depression and years of education, explaining 28% of the variance in the Block Design scores, which measure visuospatial ability, *R*=.58, *Adjusted R^2^*=.28. More years of education (ß=.45, *p*<.001) and lower depression score (ß =-1.8, *p*>.05) predicted higher visuospatial ability. When the interaction between OSA condition and education was added in the second block, the interaction term explained an additional four percent of the variance in the Block Design score, *F*(4,96)=12.17, *p*<.001 (*R^2^*_change_=.04, *p*=.019). Patients with more years of education performed better than controls as well as patients with fewer years of education (Figure 1).

**Semantic fluency:** in the first regression model, education, gender, diabetes, depression, hypertension, and condition were included as they significantly correlated with semantic fluency. By running a backward method regression, the variable condition was removed. Model 2 with five predictors explained 13% of the variance in semantic fluency. The interaction between OSA condition and education added a 3% increase in adjusted R^2^ (*R*=.46, *Adjusted R^2^*=.16, *F*(6,88)=4.01, *p*=.001). Higher education (ß =.32, *p*=.033, lower depression (ß =-.11, *p*>.05), being female (ß =-.16, *p*>.05), not having diabetes (ß =-.14, *p*>.05), and the interaction term (ß =-.29, *p*=.04) predicted 16% of the variance in semantic fluency (see Table 4.0). When patients had higher education, they performed better than controls and better than patients with fewer years of education. Patients with a high school degree or lower performed worse than controls. Hence, the effect of education on semantic verbal fluency test scores depend on the OSA syndrome. Five participants had missing data for this variable and four participants’ scores were omitted because they were higher than two standard deviations.

**Figure 2:**
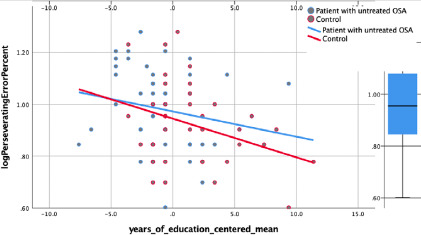
Nonsignificant interaction for executive functions (both PSQI and OSA condition were significant predictors).

**Executive functions (Wisconsin Card Sorting test):** the distribution of the number of perseverating errors on the Wisconsin Card test was positively skewed, so data transformation (i.e., logarithmic transformation) was applied. The transformed outcome variable was used in this model. Using the backward method, the multiple regression included education, mood disturbance, diabetes, and hypertension as predictor variables in Model 1. The best model (Model 2) consisted of education, hypertension, and diabetes explaining 10% of the variance in the perseverating errors on the Wisconsin Card Sorting test, which measures executive functioning in the form of mental flexibility, *R*=.36, *Adjusted R^2^*=.10, *F*(3,83)=4.22, *p*=.008. Fewer years of education (ß =-.14, *p*>.05), diagnosis of diabetes (ß=.14, *p*>.05), and hypertension (ß =.28, *p*=.007) predicted higher perseverating errors. When the interaction was added in the second block, the interaction did not contribute toward the model (*R^2^*_change_= .004, *p*>.05). Four participants did not complete the WCST due to a computer software issue and five patients’ data whose errors were higher than two standard deviations from the mean were excluded from the analysis. It is concluded that association between education and executive function does not depend on the OSA syndrome (Figure 2).

**Table 5:** The Backward Elimination Method and Hierarchical Multiple Regression to predict Semantic Fluency.

N=94	Model 1 (Backward Elimination Step 1)	Model 2 (Backward Elimination Step 2 with significant predictor variables	Model 3 (with interaction term)	Tolerance {VIF}
Constant	22.40 (.86)	22.13 (.58)	22.50 (.59)	
Condition	-.45 (1.03) [-.05]			
Education	.13 (.13) [.11]	.12 (.13) [.10]	.39 (.18) [.32]*	.40 ({2.51}
Diabetes	-1.79 (1.52) [-1.3]			
	-1.7 (.50) [-.12]	-1.9 (1.47) [-.14]	.77 {1.3}	
Depression	-.07 (.05) [-.15]	-.06 (.05) [-.14]	-.05 (.05) [-.11]	.77 {1.3}
Gender	-1.62 (.93) [-.18]	-1.51 (.89) [-.17]	-1.40 (.88) [-.16]	.94 {1.06}
Hypertension	-1.61 (1.08) [-1.8]	-1.55 (1.06) [-.17]	-1.48 (1.04) [-.16]	.66 {1.51}
Interaction (Education x Condition)			.52 (.25) [.29]*	.45 {2.22}
Adjusted R2	.12	.13	.16*	

Unstandardized regression coefficients; Standard Error (in parentheses); Standardized regression coefficients [in brackets]. N= 101.

**Psychomotor vigilance test (PVT; choice reaction time task):** the distribution of the accuracy percentage on the psychomotor vigilance test was positively skewed, so a square root transformation was applied. In the first model, the variables that significantly correlated with the accuracy percentage on the PVT include global PSQI score, diabetes, hypertension, mood disturbance, anxiety, and depression. Based on the backward method in the regression model, the second model retained global PSQI score, diabetes, and years of formal education as significant predictors, *R*=.38, *Adjusted R^2^*=.13. The interaction term was added in the second block of Model 3 using the enter method (the first block contained the variables from Model 2 using the enter method). F change was not statistically significant, meaning the interaction was not helpful for the model (*R^2^_change_*=.02, *p*>.05). Higher education (ß =-.40, *p*=.007), not having diabetes (ß =.17, *p*>.05), and lower global PSQI score which indicates better subjective sleep quality (ß =.17, *p*>.05) predicted better performance on the PVT. The four predictors combined explained 13% of the variance in vigilance or sustained attention and the interaction term did not account for more variance, *F*(4,94)=4.76, *p*=.002. Six participants had missing data for this variable and five participants had scores 2 standard deviations away from the mean.

**Attention span (WAIS III Total Digit Span):** hierarchical regression model was ran directly from the three predictor variables that shared covariation with the total digit span score in the correlational analysis. They were POMS score (mood disturbance), years of education, and anxiety. These three predictors explained 11% of the variance in total digit span score, which measures attention or concentration, *R*=.35, *Adjusted R^2^*=.11. Higher education (ß =.17, *p*>.05), lower mood disturbance (ß =-.11, *p*>.05), and lower anxiety (ß =.05, *p*>.05) predicted better attention span. Adding the interaction term in the second block did not produce significant R^2^ change (*R^2^_change_*=.01, *p*>.05), but the model was statistically significant, *F*(4,99)=4.30, *p*=.003. Seven participants had missing data for this cognitive test.

**Verbal Fluency (Phonemic fluency):** the variables that significantly correlated with phonemic fluency, namely education, mood disturbance, hypertension, condition, and depression were included in Model 1. Based on the backward method, the best model (Model 2) consisted of education and depression as predictor variables, explaining 12% of the variance in phonemic verbal fluency score, *R*=.38, *Adjusted R^2^*=.12, *F*(2,92)=7.59, *p*=.001. Higher years of education (ß =-.17, *p*>.05) and lower depression score (ß =-.19, *p*>.05) predicted higher verbal fluency. When the interaction between OSA condition and education was added in the second block of the hierarchical regression model (Model 3), the interaction term did not contribute toward the model (*R^2^*_change_=.006, *p*>.05) but the model was still statistically significant, *F*(4,99)=4.30, *p*=.003. The association between education and phonemic verbal fluency does not depend on the OSA syndrome. Six participants had missing data for this cognitive test.

## DISCUSSION

The goal of the current study was to examine the role of education on the relationship between OSA and cognitive functions. The interaction between OSA and education was examined as a moderating variable for six cognitive test scores. The interaction term was not statistically significant for all cognitive functions. The association between the cognitive performance and years of formal education plays out differently depending on the OSA vs. control condition for only visuospatial performance and semantic fluency.

For visuospatial ability, the interaction between OSA and education was still significant even after controlling for education and depression. Results indicated higher education and lower depression scores were associated with higher Block Design scores indicative of better visuospatial ability. While these predictor variables explained 28% of the variance, the moderation effect of OSA explained an additional 3%. How does education affect the two conditions differently? In order to understand this more clearly, a simple regression was conducted. It appears that for every additional year of education, the block design score increased by 0.35 standard deviation for the patients and by 0.59 standard deviation for the controls. Education seems to enhance controls’ performance twice as much as it does for patients. Nevertheless, patients with more years of education did significantly better than patients and controls with fewer years of education. Controls with higher education performed the best, followed by patients with higher education. It is likely that education serves as a protective factor for said cognitive task but since OSA was potentially taxing the cognitive load, the patients may not have had as much of a buffer from education as did the controls. The regression slope of the patient group was just not as steep as that of the control group (see Figure1).

**Figure 3:**
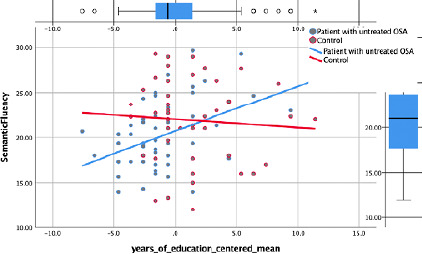
Significant interaction for semantic fluency.

Semantic fluency is another outcome variable in which we found a statistically significant interaction when depression, gender, hypertension, diabetes, and years of education were statistically held constant. Lower depression scores, being female, higher education, and not having diabetes were related to better semantic fluency. More importantly, the interaction between OSA and education explained an additional 3% of the variance in semantic fluency. The simple regression model, which was run just to help explain the interaction shows that for controls, with every additional year of education, semantic fluency score barely changed (just 0.07 standard deviation), whereas for patients, the semantic fluency score rose by 0.41 standard deviation (Figure 3). When the patients had at least a college degree, they performed better than the patients did with fewer years of education and better than the controls did (regardless of educational level). It seems that the benefit of higher education serves as a protective factor more for patients with OSA; it does not seem to enhance semantic fluency if one does not have moderate or severe OSA (mild cases were not included in the interpretation because type III sleep monitors may have missed borderline or mild OSA among the controls).

The perseverating errors made on the Wisconsin Card Sorting test were considered the measure of executive functioning. It appears that for every additional year of education, perseverating errors decreased by 0.25 standard deviations while statistically controlling for hypertension and the interaction. Although the interaction between OSA and education was not significant, education and hypertension predicted perseverating errors on the WCST and accounted for 13% of the variance in perseverating errors. This indicates that those who had a higher level of education performed better than those with fewer years of education regardless of OSA syndrome. The effect of education on executive functioning was not moderated by OSA condition.

As for attention span, phonemic fluency, and vigilance, education was the main predictor regardless of OSA condition. For instance, when a participant had an additional year of formal education, they increased .30 standard deviation in their digit span score after accounting for anxiety and mood disturbance, .26 standard deviation in phonemic fluency when depression score was constant, and .25 standard deviation in vigilance when PSQI and diabetes were held constant. However, when the interaction term was added to the models, none of the predictor variables were statistically significant. OSA condition did not moderate the relation between education and these three cognitive measures.

Higher education is regarded as one of the key factors related to higher cognitive reserve (i.e., higher resilience to neuropathological damage and/or ability to maximize performance)^9, 10^. Consistent with Alachantis et al. (2005)^9^ findings, we were able to demonstrate the benefits of education among patients with OSA on semantic fluency and visuospatial ability. The results of the current study provide evidence for resource substitution theory^6^ because when patients with OSA had more years of education, they performed as well as control participants with fewer years of education on the visuospatial block design test (but not as well as the controls with higher education). However, for semantic fluency, the fact that the control group performed similarly, regardless of educational level, provides further evidence for the resource substitution theory as education compensates for background disadvantages only in patients with untreated OSA but not in the controls. The regression line for the control group was flatter than that of the patients for semantic fluency.

An interpretation for why the statistical significance in the interaction effect was observed only for semantic fluency and visuospatial ability but not for attention (total digit span and psychomotor vigilance) is that attentional control deficits which are purportedly associated with sleep fragmentation can be compensated by higher education (hence the main effect of education for psychomotor vigilance and digit span) regardless of the OSA diagnosis. But when the cognitive demand is higher than attentional tasks but not as high as executive functions tasks (as in visuospatial ability test and semantic fluency), the main effect of education alone was not observed but a significant interaction effect, indicating that the benefit of education was more useful for patients with OSA than for controls. In addition, Kahneman (1973)^32^ attention theory states that our cognitive resource capacity is limited. Therefore, for a more rudimentary cognitive task such as sustained attention, the moderating effect of OSA was not observed. However, when the task becomes more demanding, then the compensatory mechanism of education comes into play (especially for patients with OSA^33^).

Why did we find significance with semantic fluency but not with phonemic fluency? Semantic fluency task requires participants to search through semantic memory, which is memory of facts and details required via conscious learning, whereas phonemic fluency is a search through phonological memory (i.e., sound based). Interestingly, Biesbroek et al. (2016)^34^ found evidence in brain imaging that semantic fluency may reflect the application of visuospatial mental imagery strategy as the right inferior frontal gyrus (which does spatial tasks) was involved in addition to the left inferior frontal gyrus (which does verbal tasks). Therefore, semantic fluency may be more similar to the Block design task which assesses visuospatial ability. Similarly, another study reported that age and years of education explained 42% of the variance in semantic fluency (animals) but only 26% in phonemic fluency (average of letters M, R, and P)^35^. Phonemic fluency is related to frontal lobe lesions whereas semantic fluency is mediated by the temporal lobe^36^. The effects of age on phonemic fluency is also smaller than that of semantic fluency. Since these two verbal fluency tests differ in a couple of ways, it is not surprising to find significant interaction between OSA and education with semantic fluency but not with phonemic fluency.

It is important to note that the subjective sleep (measured by the Pittsburgh sleep quality index - PSQI) was a significant correlate for visuospatial test and vigilance. Hence, subjective sleep complaints (e.g., daytime sleepiness) may be related concentration and sustained attentional tasks in middle age and older adults even when an objective health measure such as the apnea-hypopnea index does not. Consistent to the previous interpretation, Risser et al. (2000)^37^ also concluded that EEG-defined attention lapses while doing a simulated driving task were not associated with overt sleep, but inattention was related to subjective sleepiness among patients with OSA. An older adult’s subjective perception of their previous night’s sleep quality may significantly affect their performance the next day independently of objective measure of OSA severity and should be further investigated. It is important for health care providers to inquire about subjective sleep quality and sleep habits, in addition to using objective health indices with middle age and older patients to have a complete picture. Health care providers should expect older OSA patients with less education to exhibit more cognitive deficits. Since 12 to 25% of patients trying out CPAP for the first time abandon treatment within three years^38^, this is helpful information to health care providers who can tailor their intervention to encourage treatment compliance and to offer strategies to counter cognitive deficits (e.g., cognitive training on updating and mental set shifting).

### Strengths and limitations

Cognitive reserve theory has not been used as a basis to study OSA research since Alachantis et al. (2005)^9^. Alachantis et al. (2005)^9^ examined OSA-related cognitive deficits based on the cognitive reserve theory, although cited multiple times, has not been replicated in a similar sample (i.e., comparison between patients with untreated OSA and controls). The hierarchical regression used in the current study allowed us to test the effect of interaction between education and OSA on various cognitive tasks. The effect of education on health has not been extensively studied. This study had an opportunity to test for the first time the two opposing theories of the effect of education on health developed by Ross and Mirowsky (2006)^6^ in the sleep medicine field.

The limitations of the current study highlight the complexity of factors that may be responsible for cognitive performance in middle-aged and later adulthood. One, the study did not have equal group size between OSA patients (n=62) and controls (n=47). In particular, the average vs. high education group sizes were not equal across the patient group and the control group. Hence, disentangling the role of cognitive reserve from the effects of OSA severity should be considered with concern. While one study claims a strong correlation between AHI from PSG and AHI from type 3 portable monitors^39^, another study posits type 3 portable monitors can better discern moderate and severe OSA than it could with mild OSA^40^. Thus, our second limitation is that controls were screened for sleep apnea using a different screening method than was used for patients. We are now fully enlightened that the AASM recommends that PSG is more sensitive than type 3 sleep studies^29^. However, this knowledge was available after this study was completed. Since there was no clear evidence until 2017 that home studies were less, we need to emphasize in our interpretation to exclude controls with potential mild OSA. Nevertheless, it is worth reiterating that none of the controls had any clinical sleep apnea symptoms. It would have been ideal if both groups had undergone a full sleep study, but that was not logistically feasible for the control participants. This was because the patients’ PSG diagnostic studies were covered by their medical insurance because of clinical symptoms and this study recruited the patients that were ready to seek treatment at a sleep clinic.

Lastly, the duration of having undiagnosed OSA may have an effect on whether patients exhibit cognitive deficits. It is not possible to measure how long a person has had OSA without their awareness and therefore some patients may have been suffering hypoxia longer than others. Olaithe and Bucks (2013)^41^ concluded in their meta-analyses that hypoxia/ hypercarbia might be related to cognitive deficits in memory, executive function, and language abilities because these deficits have been observed in both OSA and COPD patients. Hence, the duration of patients experiencing hypoxia may influence the extent of their cognitive deficit. There is a need for longitudinal studies to identify those at risk and health care providers should look at those risk factors once they are better documented.

### Clinical implications and future directions

The intriguing question remains whether the benefits of education are masking some of the manifestation of OSA-related cognitive deficits in middle-aged and older adults, or if the benefits of education are in fact a type of compensation, where the brain is using an alternative cognitive strategy to maintain the acceptable level of performance. The scaffolding theory of aging and cognition^15^ indicates that an adaptive brain may engage in compensatory scaffolding in response to the challenges posed by declining neural structures and function (e.g., drop in blood oxygen level in OSA patients due to upper airway collapsing). We are proposing that scaffolding is protective of cognitive function in the aging brain, and the current study’s evidence suggests that the ability to use this mechanism (i.e., scaffolding) is strengthened by a higher level of education. This interpretation is in line with ReuterLorenz and Park (2014)^15^ revised STAC model where they argued that higher education may serve as life course enrichment factor in addition to neural resource enrichment (e.g., leisure activity) in assisting older adults maintain their cognitive functioning at an optimal level. Those with more education may be “rewiring” their brains better than those with less education. Hence, OSA may affect cognition adversely, but it can be kept in mind that the effect may be less evident in more educated patients. Education may “buffer” the negative effects in OSA patients especially in middle and late adulthood. The more educated patients may have subtle cognitive deficits and therefore their improvement in subjective sleep quality from using CPAP may also be subtle.

OSA patients with higher levels of education (reflective of cognitive reserve) may engage in bilateral brain activation to maintain a higher level of visuospatial ability. Based on the hemispheric asymmetry reduction in older adults (HAROLD) model, brain activation will become more generalized as we age^42^. For instance, older adults’ brain activation is more dedifferentiated and more bilateral activation is present, while young adults exhibit more unilateral activation. The challenge of future research studies is to examine more specifically how education works to support cognitive functions, to examine more specifically, what compensatory mechanisms are in place, and to determine the threshold at which education will no longer serve as a protective factor especially in clinical populations such as older patients with OSA.

Future studies examining the relations between OSA and education on cognitive functions should include additional variables such as hobbies, social life, and activities of daily living as assessed by the cognitive reserve scale^43^. Health care providers should expect older OSA patients with higher education to exhibit fewer cognitive deficits than patients with average education regardless of OSA severity. In sum, the current findings highlight the need for healthcare professionals to consider educational level when working with middle age and older patients in both the assessment and development of treatment plans.
